# Species barriers in AAV tropism: mechanisms, models, and emerging solutions for clinical translation

**DOI:** 10.3389/abp.2026.15939

**Published:** 2026-03-26

**Authors:** Xinyuan Xu

**Affiliations:** Postdoctoral Research Workstation, Beijing State-owned Capital Operation and Management Company Limited, Beijing, China

**Keywords:** AAV, AI, gene therapy, species difference, tropism

## Abstract

Adeno-associated virus (AAV) vectors have become central to *in vivo* gene therapy across genetic and acquired diseases. Yet despite extensive preclinical validation, AAV programs often encounter translational gaps when advancing from rodents to non-human primates (NHPs) and ultimately to humans. These species barriers arise from differences in capsid–receptor interactions, intracellular trafficking, immune landscapes, and tissue microanatomy. Even within species, strain-level genetic variation can markedly alter vector performance, exemplified by the LY6A-dependent central nervous system (CNS) tropism of AAV-PhP.B in C57BL/6J mice. Old world monkeys, which are evolutionarily closer to humans than new world species, remain the most widely used models for systemic and CNS delivery, yet discrepancies in seroprevalence, complement activity, endothelial biology, and neuronal susceptibility still limit predictability. Recent advances, including machine learning–guided capsid design, deep mutational scanning, and human-derived organoids and explant models, offer powerful tools to bridge these barriers. This mini-review synthesizes current understanding of AAV species barriers and outlines strategies that enhance the robustness and human relevance of AAV gene therapy development.

## Introduction

Recombinant adeno-associated virus (AAV) vectors have enabled efficient, durable *in vivo* gene transfer in numerous therapeutic areas, with clinical successes in inherited retinal diseases, spinal muscular atrophy, and hemophilia (Shen et al., 2022; Wang et al., 2019). AAV vector can be used in the context of gene replacement (Mendell et al., 2017), RNA interference (Amado et al., 2025), or gene editing (Ou et al., 2019). Despite favorable safety profiles and scalable manufacturing (Liu et al., 2024), AAV translation remains challenging because vector behavior differs substantially across species. Rodents are indispensable for discovery and proof-of-concept studies, yet their immune systems, receptor repertoires, and tissue architecture often diverge from those of non-human primates (NHPs) and humans (Byrne et al., 2025). Even among non-human primates, patterns of AAV susceptibility, tissue tropism, and biodistribution vary by clade: engineered AAV variants can show neuron-biased transduction in infant Old World monkeys yet vasculature-biased transduction in adult New World marmosets, highlighting clade-specific differences in how AAV vectors interact with host biology (Chuapoco et al., 2023).

Species barriers manifest at multiple levels: capsid binding to glycans or protein receptors, intracellular processing and innate sensing, vector dissemination across tissue barriers, and adaptive immune responses that differ in magnitude and specificity (Cao et al., 2024; Wang et al., 2024; Keeler et al., 2025). Importantly, such effects are not isolated phenomena but recur across tissues and vector classes. A prominent example is the strain-dependent enhancement of central nervous system transduction by AAV-PhP.B, which depends on LY6A expression in select C57BL/6J mouse strains and fails in other strains and in NHPs (Deverman et al., 2016; Hordeaux et al., 2018; Huang et al., 2019). In the liver, the capsid AAV-LK03 exhibits efficient transduction of human hepatocytes yet markedly reduced activity in murine livers, revealing fundamental interspecies differences in hepatocyte entry and intracellular processing (Lisowski et al., 2014). Similarly, muscle-directed capsids such as MyoAAV-1A and MyoAAV-2A demonstrate robust performance in mice but altered or diminished transduction profiles in NHPs, underscoring limitations in extrapolating muscle tropism across species (Tabebordbar et al., 2021). At a systemic level, whole-body biodistribution studies further show that AAV tropism and clearance patterns following intravenous delivery differ substantially between mice and NHPs, affecting both target engagement and off-target exposure (Fang et al., 2025).

This review summarizes molecular and physiological determinants of AAV species barriers, strain and species differences *in vivo*, and strategies—including machine-learning-guided capsid design and advanced human-derived models—that enhance translational predictability.

## Mechanistic basis of species barriers across the AAV life cycle

### Capsid architectural determinants of AAV tropism

AAV tropism is fundamentally rooted in capsid architecture. Although AAV serotypes share a conserved β-barrel core, relatively small sequence differences—particularly within surface-exposed variable loops surrounding the threefold and fivefold symmetry axes—produce substantial changes in capsid topology, electrostatics, and flexibility (Wang et al., 2019). These architectural features establish the physical framework that governs how each serotype interacts with host factors. Canonical serotypes illustrate this principle. AAV2 and AAV9, despite overall structural similarity, differ markedly in surface charge distribution and loop conformation, creating distinct binding interfaces and baseline tropism profiles (Wu et al., 2006; Wang et al., 2019). Importantly, these architectural differences precede and constrain receptor usage rather than arising from it.

### Receptor and glycan engagement as a consequence of capsid architecture

Receptor and glycan engagement are direct functional consequences of capsid structure. AAV2 binds heparan sulfate proteoglycans through a basic surface motif, whereas AAV9 engages terminal galactose residues via a structurally distinct capsid pocket (Romanovsky et al., 2025; Hoffman et al., 2024). These interactions arise from serotype-specific surface chemistry encoded by capsid architecture, rather than from receptor biology alone. Importantly, the functional outcome of these capsid–glycan interactions varies across species, as glycan abundance, structure, and tissue distribution differ between rodents, non-human primates, and humans, thereby modulating AAV binding avidity and downstream tropism (Hoffman et al., 2024). This structure–function relationship explains why modest capsid sequence changes can reprogram receptor usage and tropism. Both natural serotype divergence and engineered modifications of surface loops have been shown to alter attachment factors and downstream biodistribution, reinforcing the central role of capsid architecture in shaping vector behavior (Cabanes-Creus et al., 2021; Wang et al., 2019).

### Species-specific receptor landscapes and post-attachment processing

While capsid architecture defines the potential for receptor engagement, the realized tropism of an AAV vector depends on species-specific biology. Expression patterns of glycans and protein receptors differ across rodents, non-human primates, and humans, leading to divergent transduction efficiencies even for identical capsids (Lisowski et al., 2014; Westhaus et al., 2023). Beyond attachment, species differences in endocytic trafficking, endosomal escape, nuclear entry, and innate immune sensing further modulate vector performance (Wang et al., 2019). Unmethylated CpG motifs within the AAV genome activate TLR9 signaling in plasmacytoid dendritic cells, and the magnitude of this response varies across species and human donors, influencing early innate activation and vector clearance (Alakhras et al., 2024; Suriano et al., 2024). In addition, depending on serotypes, different levels of pre-existing antibodies exist in human populations (Boutin et al., 2010), which significantly affects transduction and tissue tropism (Meliani et al., 2015; Schulz et al., 2023).

## Strain and species differences *in vivo*

Rodent strains exhibit profound differences in susceptibility to individual AAV variants. The most striking case is PhP.B, which provides high-level central nervous system (CNS) transduction in C57BL/6J mice following intravenous injection but fails almost entirely in BALB/c mice, other mouse strains, NHPs, and humans (Deverman et al., 2016; Hordeaux et al., 2018; Huang et al., 2019; Matsuzaki et al., 2019). This phenotype depends on expression of LY6A on brain endothelium, a protein absent from many mouse strains and primates. PhP.B thus illustrates how strain-specific genetics can create misleading impressions of cross-species transduction potential. Beyond the CNS, hepatic and immune traits also vary among rodent strains. Differences in Kupffer cell abundance, complement activation, and sinusoidal fenestration could influence vector uptake and clearance.

NHPs provide a closer physiological and immunological approximation to humans than rodents and are therefore central to translational AAV development. Old World monkeys, particularly rhesus and cynomolgus macaques, are widely used for systemic and CNS studies because key aspects of their glycan landscapes, complement systems, and immune repertoires more closely parallel those of humans (High and Roncarolo, 2019). Nevertheless, even within macaques, inter-colony variability in pre-existing immunity remains substantial. Neutralizing antibody titers against AAV1, AAV2, AAV8, and AAV9 can range from undetectable to high depending on geographic origin, husbandry, and viral exposure history (Boutin et al., 2010), complicating cross-study comparisons and translational interpretation. New World monkeys, such as the common marmoset, are also widely used in AAV research and often reproduce major features of vector biodistribution observed in macaques, particularly for CNS- and vasculature-targeting capsids. However, marmosets possess distinct immunogenetic and developmental features that can influence immune-mediated readouts and vector interpretation. These include reduced diversity and structural differences in major histocompatibility complex (MHC) haplotypes, frequent hematopoietic chimerism, and differences in endothelial and neurodevelopmental biology relative to Old World primates and humans (Antunes et al., 1998; Sweeney et al., 2012; Seki et al., 2017). Consistent with this, engineered AAVs can show species- and age-dependent shifts in CNS cell-type bias between marmosets and macaques (Chuapoco et al., 2023). As a result, while marmosets are valuable for assessing feasibility, developmental neurobiology, and ophthalmic delivery, caution is warranted when extrapolating performance directly to humans.

Human clinical data underscore the inadequacy of rodent-only and even NHP-only predictions. Pre-existing neutralizing antibodies remain a major barrier to effective AAV transduction across indications (Li et al., 2022). High-dose intravenous AAV9-like vectors have caused severe hepatotoxicity and dorsal root ganglion injury in NHPs and piglets, phenomena not fully anticipated by rodent studies (Hinderer et al., 2018). MicroRNA-regulated expression systems can mitigate these toxicities in NHPs (Hordeaux et al., 2020), illustrating how species-appropriate refinements can directly improve safety.

## Tissue- and structure-level barriers

Beyond molecular determinants, tissue microanatomy strongly influences vector performance. In the retina, the inner limiting membrane (ILM) is considerably thicker and structurally more complex in NHPs and humans than in mice. Dalkara and colleagues showed that many capsids able to cross the murine ILM and transduce photoreceptors after intravitreal delivery perform poorly in larger species, necessitating subretinal delivery or specialized engineered variants (Dalkara et al., 2013). Human stem cell–derived retinal pigment epithelium and photoreceptors display distinct capsid preferences relative to murine tissues, emphasizing the need for human-relevant models (Gonzalez-Cordero et al., 2018). In the liver, sinusoidal endothelial cell fenestration and scavenger endothelial cell biology differ markedly across species (Shetty et al., 2018). These differences affect not only hepatocyte access but also opsonization and sequestration by non-parenchymal cells. The blood–brain barrier (BBB) also differs structurally between rodents and primates. Rodent endothelial cells possess distinct transporter and glycocalyx profiles compared with macaques and humans, contributing to discrepancies in CNS penetration of systemically delivered AAV. PhP.B’s failure to cross the primate BBB illustrates this point clearly (Hordeaux et al., 2018). Age and disease state further modulate BBB permeability, potentially affecting AAV CNS tropism (Foust et al., 2009). These structural differences highlight the need for model systems that recapitulate human tissue barriers, particularly when vectors are intended for intravitreal, intrathecal, or systemic delivery.

## Emerging approaches to overcome species barriers

### Receptor-defined capsid engineering to overcome species-specific tropism

A major source of species divergence arises at the level of capsid–receptor compatibility, where capsids selected in rodents often rely on receptors that are absent or functionally divergent in primates and humans. Recent capsid engineering efforts have therefore shifted toward receptor-defined strategies that emphasize evolutionary conservation, directly addressing this long-standing barrier (Table 1). A prominent example is VCAP-102, which exploits alkaline phosphatase (ALPL), a brain vascular receptor that is highly conserved across rodents, non-human primates, and humans (Moyer et al., 2025). Engagement of ALPL enables robust CNS gene transfer following systemic delivery in both mice and cynomolgus macaques, while limiting peripheral transduction relative to AAV9-like vectors. Importantly, the translational relevance of VCAP-102 derives from its reliance on a conserved endothelial receptor, thereby reducing the strain- and species-specific artifacts that have historically limited predictive power in AAV preclinical studies. A complementary paradigm is illustrated by AAV-hCA4-IV77, 9P31, and 9P36, which target carbonic anhydrase IV (CA4) on brain microvascular endothelium. CA4 expression is conserved between rodents and primates, and these capsids demonstrate efficient blood–brain barrier transcytosis after intravenous administration in mouse, marmoset, and macaque models, with reduced liver tropism compared with AAV9 (Shay et al., 2023; Lin et al., 2025). Validation of CA4 binding in human endothelial cells and humanized receptor systems further supports the clinical relevance of this pathway. These vectors stand in sharp contrast to murine-restricted capsids such as AAV-PhP.B, whose dependence on LY6A explains their failure outside select mouse strains and in non-human primates (Huang et al., 2019; Hordeaux et al., 2018). Collectively, these examples highlight a broader shift in AAV engineering: robust clinical translation increasingly depends on targeting receptors with conserved vascular and cellular biology, rather than optimizing performance in a single preclinical species.

**TABLE 1:** Receptors and tissue tropism of mainstream AAV serotypes.

Capsid	Primary receptors	Main tissue tropism	Main species/models where shown	Refs
AAV1	AAVR; α2,3/α2,6 N-linked sialic acid on glycoproteins	Skeletal and cardiac muscle (strong), also liver in some settings	Mouse, rat	Wu et al. (2006); Zincarelli et al. (2008); Pillay et al. (2016)
AAV2	AAVR; heparan sulfate proteoglycan (HSPG)	Retina (inner retina, Müller/glia) after intravitreal injection; photoreceptors/RPE after subretinal injection; liver and CNS in some models	Mouse, rat, dog, NHP; human (ocular and liver gene therapy trials)	Wu et al. (2006)
AAV5	AAVR; α2,3-linked sialic acid; PDGFR	Airway epithelium, CNS, retina (especially via subretinal delivery), some liver tropism	Mouse, ferret, NHP	Wu et al. (2006); Pillay et al. (2017)
AAV7	AAVR; O-linked glycans/gangliosides	Liver and retina (subretinal) with strong, long-term photoreceptor and RPE transduction	Mouse, dog	Wu et al. (2006); Pillay et al. (2016)
AAV8	AAVR; laminin receptor	Liver (very strong hepatotropism), also heart, skeletal muscle; efficient retina transduction after subretinal injection	Mouse, NHP; human	Gao et al. (2002); Zincarelli et al. (2008); Nathwani et al. (2011)
AAV9	AAVR; terminal galactose; laminin receptor	CNS (crosses BBB after IV in rodents), heart, skeletal muscle, liver; widely used for systemic CNS and muscle delivery	Mouse, rat, dog, NHP; human (e.g., Zolgensma)	Zincarelli et al. (2008); Shen et al. (2011); Mendell et al. (2017)
AAVrh10	AAVR; terminal galactose	CNS (brain, spinal cord, PNS) and liver after IV, strong spinal cord transduction in neonatal mice; used for Krabbe disease and other CNS disorders	Mouse, NHP; early human Krabbe trials	Tanguy et al. (2015); Mietzsch et al. (2021)
AAV11, AAV12	AAVR2/CPD (carboxypeptidase D)	Broad tropism in cultured cells and mouse tissues when CPD is expressed; clade E-like vectors with robust liver and CNS transduction in preclinical models	Human cells, mouse; structural work also suggests compatibility with human tissues	Dhungel et al. (2025)
AAV-hCA4-IV77, 9P31, 9P36	Carbonic anhydrase IV (CA4) on brain microvascular endothelium; CA4 expression is conserved between mice and primates	Efficient BBB crossing and CNS transduction after IV; reduced liver transduction compared with AAV9 in primate-relevant models	Mouse, marmoset, macaque; human CA4-binding variants validated in human endothelial cells and humanized receptor models	Shay et al. (2023); Lin et al. (2025)
VCAP-102	ALPL; ALPL expression is highly conserved in brain vasculature across rodents and primates	Robust CNS gene transfer after IV with limited peripheral transduction; strong vascular and parenchymal CNS expression	Mouse and NHP (cynomolgus macaque); human endothelial cells *in vitro*	Moyer et al. (2025)
PhP.B, PhP.eB	LY6A (Sca-1) on mouse endothelial cells; LY6A expression pattern is strain- and species-restricted (prominent in C57BL/6J, absent in many other strains and in primates)	Dramatic pan-CNS transduction after IV in LY6A-positive mouse strains; no enhanced CNS tropism in most other mouse strains or in NHPs	Mouse (C57BL/6J and related); negative results in BALB/c and NHP highlight species/strain barrier	Huang et al. (2019); Hordeaux et al. (2018)
AAV9-X1.1, CAP-Mac, PhP.eC	LRP6; LRP6 is a conserved receptor, but the engineered capsid–LRP6 interaction has been validated mainly in human cells and mice	Enhanced CNS transduction after IV with reduced peripheral expression in engineered variants; human-cell–derived interactome suggests relevance for primate BBB	Human brain endothelial cells (*in vitro*), mouse; interactome data from human cell lines	Shay et al. (2024)
BI-hTFR1 family	Human transferrin receptor 1 (TfR1/TFRC); receptor is conserved, but these capsids bind specifically to human TfR1, with enhanced CNS delivery only in knock-in mice expressing human TFRC	Brain-wide CNS delivery after IV in human TFRC knock-in mice; no enhanced tropism in wildtype mice or NHPs	Human TFRC knock-in mice; human endothelial cells *in vitro*; no CNS gain-of-function in standard mouse or NHP	Huang et al. (2024)

AAVR, adeno-associated virus receptor.

### ML-guided and directed evolution approaches as tools to resolve species mismatch

Machine learning–guided capsid design and next-generation directed evolution platforms are increasingly used to identify capsid features that generalize across species, rather than those that overfit rodent-specific biology (Ghauri and Ou, 2023). When trained on datasets derived from primate or human-relevant systems, these approaches can uncover structural motifs associated with conserved receptor engagement, intracellular trafficking, or immune evasion. Crucially, the value of ML-guided approaches lies not in their computational novelty, but in their ability to systematically interrogate capsid–host interactions across species, accelerating discovery of capsids that maintain function in primate and human contexts (Ogden et al., 2019; Bryant et al., 2021). When coupled to appropriate selection environments, these tools directly address barriers arising from species-specific receptor usage and post-entry processing.

### Iterative cross-species evaluation and using humanized and xenotransplant models

In practice, a key strategy for overcoming species barriers is the iterative testing of the same AAV vector across multiple species, rather than sequential optimization in a single model. Parallel evaluation in rodents, non-human primates, and human-relevant systems enables early identification of failures in receptor usage, intracellular trafficking, immune recognition, or clearance that would otherwise be masked by species-specific biology. This pragmatic approach reduces overfitting to rodent models and has become a central component of translational AAV development pipelines, particularly for vectors intended for systemic delivery or clinical dose escalation (Gonzalez et al., 2022; Westhaus et al., 2023).

Even when capsid–receptor interactions are conserved, species barriers frequently emerge from differences in intracellular trafficking, innate immune sensing, and clearance pathways. Humanized mouse models and hepatocyte or endothelial xenotransplant systems address this challenge by providing human cellular substrates within an *in vivo* context, thereby revealing failures that are masked in conventional rodent models (Lisowski et al., 2014). These systems function as mechanistic filters rather than predictive surrogates: capsids that perform well in rodents but poorly in humanized models can be deprioritized early, while vectors that retain activity are more likely to overcome downstream species barriers (Ghauri and Ou, 2023). In this way, humanized models directly address limitations in translational predictability rather than merely improving model sophistication.

In addition, neutralizing antibodies (NAbs) represent an additional species- and individual-dependent barrier that complicates cross-species comparisons. Beyond prescreening for pre-existing antibodies, immunodeficient animal models are commonly used to isolate intrinsic capsid tropism and biodistribution from antibody-mediated effects, enabling mechanistic evaluation of vector performance in the absence of adaptive immunity (Mingozzi and High, 2013). In larger-animal and translational studies, transient immunosuppression, including corticosteroid-based or targeted regimens, has been employed to attenuate humoral and cellular immune responses, improving vector persistence and transgene expression while allowing assessment of non-immune species barriers (Li et al., 2022; Keeler et al., 2025). Together, these approaches provide complementary tools for disentangling immune-mediated effects from capsid- and tissue-intrinsic determinants of AAV transduction.

### Organoid and multicellular systems to address tissue-level species barriers

Species barriers also arise from differences in tissue architecture, multicellular organization, and dose-dependent responses that cannot be captured by monocultures or simplified *in vivo* systems. Human organoid platforms—particularly liver and brain vascular organoids (Berreur et al., 2025)—provide a means to interrogate AAV performance within human-specific microenvironments, including endothelial–parenchymal interactions and clearance mechanisms (Gonzalez-Cordero et al., 2018; Cho et al., 2021). By preserving aspects of human tissue organization and enabling controlled dose–response studies, organoids address species barriers linked to vector dissemination, sequestration, and toxicity, complementing both *in vivo* primate studies and receptor-centric capsid engineering efforts.

## Discussion

Despite significant advances in capsid engineering and human-relevant model systems, species barriers remain a major limitation in AAV gene therapy. Several key challenges remain unresolved. First, receptor conservation alone does not ensure translational success. While receptor-defined capsids improve cross-species performance, downstream processes such as intracellular trafficking, uncoating, and nuclear entry remain poorly understood and may differ substantially between species. Clarifying these post-entry mechanisms in human-relevant systems remains a critical need. Second, immune-mediated species differences are difficult to predict and model. Variability in innate sensing, complement activation, and pre-existing humoral immunity can strongly influence vector clearance and toxicity (Li et al., 2022), yet these responses differ across species and even among non-human primates. Improved immune-competent humanized systems and standardized immunoprofiling will be essential for improving predictability. Third, dose-dependent and tissue-level effects remain underexplored. Many species barriers emerge only at clinically relevant doses, where nonlinear effects such as scavenger saturation or endothelial injury become apparent. Preclinical studies that explicitly address dose scaling are needed to bridge this gap. Finally, no single experimental model captures all relevant species barriers. Rodent, non-human primate, humanized, and organoid systems each provide partial insights, underscoring the need for integrated, multi-model evaluation strategies. Together, these limitations highlight that overcoming species barriers is a systems-level challenge that will require coordinated advances in capsid design, immune characterization, and human-relevant modeling, rather than further optimization within isolated preclinical contexts.

**FIGURE 1:**
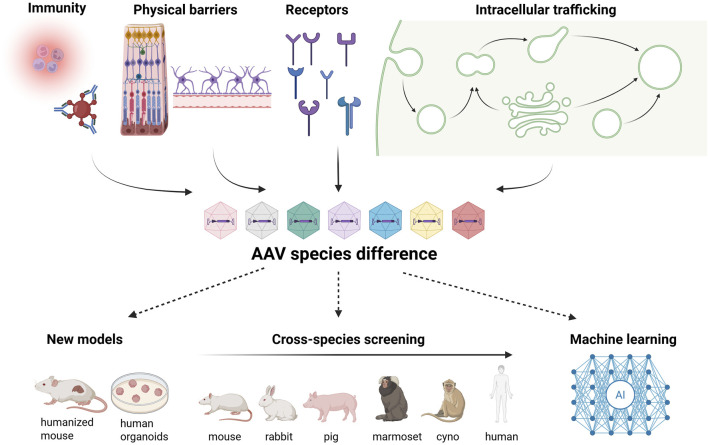
Biological determinants of AAV species differences and strategies to overcome translational barriers. Species differences in AAV transduction arise from multiple stages of the viral life cycle, including immune responses, physical tissue barriers, cell-surface receptor usage, and intracellular trafficking pathways. The top row summarizes these determinants, with arrows indicating how divergence at any stage can contribute to species-dependent differences in AAV tropism, biodistribution, and efficacy. The bottom row illustrates emerging approaches to address these barriers. Humanized and organoid-based models capture human-specific biology, cross-species screening identifies vectors that retain function across multiple animal models and humans, and machine learning–guided design integrates multi-species data to inform capsid engineering. Solid arrows denote causal influences across the AAV life cycle, while dashed arrows indicate iterative feedback between biological insights and vector design.

In summary, AAV species barriers arise from a combination of molecular, cellular, anatomical, and immunological factors that differ significantly across rodents, NHPs, and humans (Figure 1). These differences can lead to both false positives and false negatives in preclinical studies and therefore must be carefully considered during vector selection and study design. Integrating multi-species data, machine-learning-guided capsid engineering, and human organoid and explant systems offers an opportunity to improve translational reliability (Figure 1). As the field advances, embracing species and strain diversity as informative rather than inconvenient will be essential to design vectors that are safer, more predictable, and more effective for human patients.

## References

[B1] Alakhras N. S. Moreland C. A. Wong L. C. Raut P. Kamalakaran S. Wen Y. (2024). Essential role of pre-existing humoral immunity in TLR9-mediated type I IFN response to recombinant AAV vectors in human whole blood. Front. Immunol. 15, 1354055. 10.3389/fimmu.2024.1354055 39007143 PMC11240241

[B2] Amado D. A. Robbins A. B. Whiteman K. R. Smith A. R. Chillon G. Chen Y. (2025). Erratum: AAV-based delivery of RNAi targeting ataxin-2 improves survival and pathology in TDP-43 mice. Nat. Commun. 16 (1), 9356. 10.1038/s41467-025-65424-5 41131050 PMC12549958

[B3] Antunes S. G. de Groot N. G. Brok H. Doxiadis G. Menezes A. A. Otting N. (1998). The common marmoset: a new world primate species with limited MHC class II variability. Proc. Natl. Acad. Sci. U. S. A. 95 (20), 11745–11750. 10.1073/pnas.95.20.11745 9751736 PMC21711

[B4] Berreur E. Lazzaroni G. Roth C. Zihlmann M. Stirn M. Matheis R. (2025). iPSC-hepatocyte organoids as a novel platform to predict AAV gene therapy efficacy. Mol. Ther. Methods and Clin. Dev. 33 (2), 101467. 10.1016/j.omtm.2025.101467 40927767 PMC12415976

[B5] Boutin S. Monteilhet V. Veron P. Leborgne C. Benveniste O. Montus M. F. (2010). Prevalence of serum IgG and neutralizing factors against adeno-associated virus (AAV) types 1, 2, 5, 6, 8, and 9 in the healthy population. Hum. Gene Ther. 21 (6), 704–712. 10.1089/hum.2009.182 20095819

[B6] Bryant D. H. Bashir A. Sinai S. Kumar A. Ottolen S. G. Christiansen A. (2021). Deep diversification of an AAV capsid protein by machine learning. Nat. Biotechnol. 39 (6), 691–696. 10.1038/s41587-020-00793-4 33574611

[B7] Byrne B. J. Flanigan K. M. Matesanz S. E. Finkel R. S. Waldrop M. A. D’Ambrosio E. S. (2025). Current clinical applications of AAV-mediated gene therapy. Mol. Ther. 33 (6), 2479–2516. 10.1016/j.ymthe.2025.04.045 40329530 PMC12172329

[B8] Cabanes-Creus M. Navarro R. G. Zhu E. Baltazar G. Liao S. H. Y. Drouyer M. (2021). Novel human liver-tropic AAV variants define transferable domains that markedly enhance the human tropism of AAV7 and AAV8. Mol. Ther. – Methods and Clin. Dev. 24, 88–101. 10.1016/j.omtm.2021.11.011 34977275 PMC8693155

[B9] Cao D. Byrne B. J. de Jong Y. P. Terhorst C. Duan D. Herzog R. W. (2024). Innate immune sensing of adeno-associated virus vectors. Hum. Gene Ther. 35 (13–14), 451–463. 10.1089/hum.2024.040 38887999 PMC11310564

[B11] Cho A. N. Jin Y. Kim S. Choi Y. S. Lee J. Lee S. H. (2021). Microfluidic device-assisted organoid culture for efficient AAV-mediated gene delivery into human brain organoids. Adv. Sci. 8 (16), 2002542. 10.1002/advs.202002542

[B12] Chuapoco M. R. Flytzanis N. C. Goeden N. Octeau J. C. Roxas K. M. Chan K. Y. (2023). Adeno-associated viral vectors for functional intravenous gene transfer throughout the non-human primate brain. Nat. Nanotechnol. 18 (10), 1241–1251. 10.1038/s41565-023-01419-x 37430038 PMC10575780

[B13] Dalkara D. Byrne L. C. Klimczak R. R. Visel M. Yin L. Merigan W. H. (2013). In vivo–directed evolution of a new adeno-associated virus for therapeutic outer retinal gene delivery from the vitreous. Sci. Transl. Med. 5 (189), 189ra76. 10.1126/scitranslmed.3005708 23761039

[B14] Deverman B. E. Pravdo P. L. Simpson B. P. Kumar S. R. Chan K. Y. Banerjee A. (2016). Cre-dependent selection yields AAV variants for widespread gene transfer to the adult brain. Nat. Biotechnol. 34 (2), 204–209. 10.1038/nbt.3440 26829320 PMC5088052

[B15] Dhungel B. P. Xu H. Nagarajah R. Vitale J. Wong A. C. H. Gokal D. (2025). An alternate receptor for adeno-associated viruses. Cell 188 (18), 4924–4935.e23. 10.1016/j.cell.2025.06.026 40664211

[B16] Fang K. Yang X. Liu Y. Xia J. Wu R. Yang F. (2025). A comprehensive study of AAV tropism across C57BL/6 mice, BALB/c mice, and crab-eating macaques. Mol. Ther. – Methods and Clin. Dev. 33 (1), 101434. 10.1016/j.omtm.2025.101434 40104150 PMC11919325

[B17] Foust K. D. Nurre E. Montgomery C. L. Hernandez A. Chan C. M. Kaspar B. K. (2009). Intravascular AAV9 preferentially targets neonatal neurons and adult astrocytes. Nat. Biotechnol. 27 (1), 59–65. 10.1038/nbt.1515 19098898 PMC2895694

[B18] Gao G. P. Alvira M. R. Wang L. Calcedo R. Johnston J. Wilson J. M. (2002). Novel adeno-associated viruses from rhesus monkeys. Proc. Natl. Acad. Sci. U. S. A. 99 (18), 11854–11859. 10.1073/pnas.182412299 12192090 PMC129358

[B19] Ghauri M. S. Ou L. (2023). AAV engineering for improving tropism to the central nervous system. Biology 12 (2), 186. 10.3390/biology12020186 36829465 PMC9953251

[B20] Gonzalez T. J. Simon K. E. Blondel L. O. Fanous M. M. Roger A. L. Maysonet M. S. (2022). Cross-species evolution of a highly potent AAV variant for therapeutic gene transfer and genome editing. Nat. Commun. 13 (1), 5947. 10.1038/s41467-022-33745-4 36210364 PMC9548504

[B21] Gonzalez-Cordero A. West E. L. Pearson R. A. Duran Y. Carvalho L. S. Chu C. J. (2018). Assessment of AAV vector tropisms for mouse and human pluripotent stem cell-derived RPE and photoreceptor cells. Hum. Gene Ther. 29 (10), 1124–1139. 10.1089/hum.2018.027 29580100

[B22] High K. A. Roncarolo M. G. (2019). Gene therapy. N. Engl. J. Med. 381 (5), 455–464. 10.1056/NEJMra1706910 31365802

[B23] Hinderer C. Katz N. Buza E. L. Dyer C. Goode T. Bell P. (2018). Severe toxicity in nonhuman primates and piglets following high-dose intravenous AAV. Hum. Gene Ther. 29 (3), 285–298. 10.1089/hum.2018.015 29378426 PMC5865262

[B24] Hoffman J. A. Denton N. Sims J. J. Meggersee R. Zhang Z. Olagbegi K. (2024). Modulation of AAV9 galactose binding yields novel gene delivery variants with altered biodistribution. Hum. Gene Ther. 35 (13–14), 451–463. 10.1089/hum.2024.050 39001819

[B25] Hordeaux J. Wang Q. Katz N. Buza E. L. Bell P. Wilson J. M. (2018). The neurotropic properties of AAV-PHP.B are limited to C57BL/6J mice. Mol. Ther. 26 (3), 664–668. 10.1016/j.ymthe.2018.01.018 29428298 PMC5911151

[B26] Hordeaux J. Buza E. L. Jeffrey B. Song C. Jahan T. Yuan Y. (2020). MicroRNA-mediated inhibition of transgene expression reduces dorsal root ganglion toxicity by AAV vectors in primates. Sci. Transl. Med. 12 (569), eaba9188. 10.1126/scitranslmed.aba9188 33177182

[B27] Huang Q. Chan K. Y. Tobey I. G. Chan Y. A. Poterba T. Boutros C. L. (2019). Delivering genes across the blood–brain barrier: LY6A, a novel cellular receptor for AAV-PHP.B capsids. PLoS One 14 (11), e0225206. 10.1371/journal.pone.0225206 31725765 PMC6855452

[B28] Huang Q. Chan K. Y. Wu J. Botticello-Romero N. R. Zheng Q. Lou S. (2024). An AAV capsid reprogrammed to bind human transferrin receptor mediates brain-wide gene delivery. Science 384 (6701), 1220–1227. 10.1126/science.adm8386 38753766 PMC13387621

[B30] Keeler A. M. Zhan W. Ram S. Fitzgerald K. A. Gao G. (2025). The curious case of AAV immunology. Mol. Ther. 33 (5), 1946–1965. 10.1016/j.ymthe.2025.03.037 40156190 PMC12126790

[B31] Li X. Wei X. Lin J. Ou L. (2022). A versatile toolkit for overcoming AAV immunity. Front. Immunol. 13, 991832. 10.3389/fimmu.2022.991832 36119036 PMC9479010

[B32] Lin C. Chen X. Hoang J. D. Ristic F. Fan Y. Jang S. (2025). AAVs targeting human carbonic anhydrase IV enhance gene delivery to the brain. Cell Rep. 44 (11), 116419. 10.1016/j.celrep.2025.116419 41175373 PMC12942408

[B33] Lisowski L. Jang J. H. Vassallo M. Rost M. Sauter D. Mingozzi F. (2014). Selection and evaluation of clinically relevant AAV variants in a humanized liver model. Mol. Ther. 22 (4), 658–665. 10.1038/mt.2014.1

[B34] Liu S. Li J. Peraramelli S. Luo N. Chen A. Dai M. (2024). Systematic comparison of rAAV vectors manufactured using large-scale suspension cultures of Sf9 and HEK293 cells. Mol. Ther. 32 (1), 74–83. 10.1016/j.ymthe.2023.11.022 37990495 PMC10787191

[B36] Matsuzaki Y. Tanaka M. Hakoda S. Masuda T. Miyata R. Konno A. (2019). Intravenous administration of AAV-PHP.B in cynomolgus macaques: limited CNS transduction. Mol. Ther. – Methods and Clin. Dev. 13, 27–36. 10.1016/j.omtm.2019.01.010 30603655

[B37] Meliani A. Leborgne C. Triffault S. Jeanson-Leh L. Veron P. Mingozzi F. (2015). Determination of anti-adeno-associated virus vector neutralizing antibody titer with an *in vitro* reporter system. Hum. Gene Ther. Methods 26 (2), 45–53. 10.1089/hgtb.2015.037 25819687 PMC4403012

[B38] Mendell J. R. Al-Zaidy S. Shell R. Arnold W. D. Rodino-Klapac L. R. Prior T. W. (2017). Single-dose gene-replacement therapy for spinal muscular atrophy. N. Engl. J. Med. 377 (18), 1713–1722. 10.1056/NEJMoa1706198 29091557

[B39] Mietzsch M. Yu J. C. Hsi J. Chipman P. Broecker F. Fuming Z. (2021). Structural study of AAVrh.10 receptor and antibody interactions. J. Virology 95 (23), e01249. 10.1128/JVI.01249-21 34549984 PMC8577363

[B40] Mingozzi F. High K. A. (2013). Immune responses to AAV vectors. Blood 122 (1), 23–36. 10.1182/blood-2013-01-306647 23596044 PMC3701904

[B41] Moyer T. C. Hoffman B. A. Chen W. Shah I. Ren X. Q. Knox T. (2025). Highly conserved brain vascular receptor ALPL mediates transport of engineered AAV vectors across the blood–brain barrier. Mol. Ther. 33 (8), 3902–3916. 10.1016/j.ymthe.2025.04.046 40340250 PMC12461625

[B43] Nathwani A. C. Tuddenham E. G. Rangarajan S. Rosales C. McIntosh J. Linch D. C. (2011). Adenovirus-associated virus vector-mediated gene transfer in hemophilia B. N. Engl. J. Med. 365 (25), 2357–2365. 10.1056/NEJMoa1108046 22149959 PMC3265081

[B44] Ogden P. J. Kelsic E. D. Sinai S. Church G. M. (2019). Comprehensive AAV capsid fitness landscape reveals a viral gene and enables machine-guided design. Science 366 (6469), 1139–1143. 10.1126/science.aaw2900 31780559 PMC7197022

[B45] Ou L. DeKelver R. C. Rohde M. Tom S. Radeke R. St Martin S. J. (2019). ZFN-mediated *in vivo* genome editing corrects murine Hurler syndrome. Mol. Ther. 27 (1), 178–187. 10.1016/j.ymthe.2018.10.018 30528089 PMC6319315

[B46] Pillay S. Meyer N. L. Puschnik A. S. Davulcu O. Diep J. Ishikawa Y. (2016). An essential receptor for adeno-associated virus infection. Nature 530 (7588), 108–112. 10.1038/nature16465 26814968 PMC4962915

[B47] Pillay S. Zou W. Cheng F. Puschnik A. S. Meyer N. L. Ganaie S. S. (2017). Adeno-associated virus (AAV) serotypes have distinctive interactions with domains of the cellular AAV receptor. J. Virology 91 (18), e00391. 10.1128/JVI.00391-17 28679762 PMC5571256

[B50] Romanovsky D. Scherk H. Föhr B. Babutzka S. Bogedein J. Lu Y. (2025). Heparan sulfate proteoglycan affinity of adeno-associated virus vectors: implications for retinal gene delivery. Eur. J. Pharm. Sci. 206, 107012. 10.1016/j.ejps.2025.107012 39805508

[B51] Schulz M. Levy D. I. Petropoulos C. J. Bashirians G. Winburn I. Mahn M. (2023). Binding and neutralizing anti-AAV antibodies: detection and implications for rAAV-mediated gene therapy. Mol. Ther. 31 (3), 616–630. 10.1016/j.ymthe.2023.01.010 36635967 PMC10014285

[B52] Seki F. Hikishima K. Komaki Y. Hata J. Uematsu A. Okahara N. (2017). Developmental trajectories of macroanatomical structures in the common marmoset brain. Neuroscience 364, 143–156. 10.1016/j.neuroscience.2017.09.021 28939259

[B53] Shay T. F. Sullivan E. E. Ding X. Chen X. Ravindra Kumar S. Goertsen D. (2023). Primate-conserved carbonic anhydrase IV and murine-restricted LY6C1 enable blood–brain barrier crossing by engineered viral vectors. Sci. Adv. 9 (16), eadg6618. 10.1126/sciadv.adg6618 37075114 PMC10115422

[B54] Shay T. F. Jang S. Brittain T. J. Chen X. Walker B. Tebbutt C. (2024). Human cell surface–AAV interactomes identify LRP6 as blood–brain barrier transcytosis receptor and immune cytokine IL-3 as AAV9 binder. Nat. Commun. 15 (1), 7853. 10.1038/s41467-024-52149-0 39245720 PMC11381518

[B55] Shen S. Bryant K. D. Brown S. M. Randell S. H. Asokan A. (2011). Terminal N-linked galactose is the primary receptor for adeno-associated virus 9. J. Biol. Chem. 286 (15), 13532–13540. 10.1074/jbc.M110.210922 21330365 PMC3075699

[B56] Shen W. Liu S. Ou L. (2022). rAAV immunogenicity, toxicity, and durability in 255 clinical trials: a meta-analysis. Front. Immunol. 13, 1001263. 10.3389/fimmu.2022.1001263 36389770 PMC9647052

[B57] Shetty S. Lalor P. F. Adams D. H. (2018). Liver sinusoidal endothelial cells—gatekeepers of hepatic immunity. Nat. Rev. Gastroenterology and Hepatology 15 (9), 555–567. 10.1038/s41575-018-0020-y 29844586 PMC7096836

[B59] Suriano C. M. Kumar N. Verpeut J. L. Ma J. Jung C. Dunn C. E. (2024). An innate immune response to adeno-associated virus genomes decreases cortical dendritic complexity and disrupts synaptic transmission. Mol. Ther. 32 (6), 1721–1738. 10.1016/j.ymthe.2024.03.036 38566414 PMC11184335

[B60] Sweeney C. G. Curran E. Westmoreland S. V. Mansfield K. G. Vallender E. J. (2012). Quantitative molecular assessment of chimerism across tissues in marmosets and tamarins. BMC Genomics 13, 98. 10.1186/1471-2164-13-98 22429831 PMC3337283

[B61] Tabebordbar M. Lagerborg K. A. Stanton A. King E. M. Ye S. Tellez L. (2021). Directed evolution of a family of AAV capsid variants enabling potent muscle-directed gene delivery across species. Cell 184 (19), 4919–4938.e22. 10.1016/j.cell.2021.08.028 34506722 PMC9344975

[B62] Tanguy Y. Biferi M. G. Besse A. Astord S. Cohen-Tannoudji M. Marais T. (2015). Systemic AAVrh10 provides higher transgene expression than AAV9 in the brain and the spinal cord of neonatal mice. Front. Mol. Neurosci. 8, 36. 10.3389/fnmol.2015.00036 26283910 PMC4516891

[B63] Wang D. Tai P. W. L. Gao G. (2019). Adeno-associated virus vector as a platform for gene therapy delivery. Nat. Rev. Drug Discov. 18 (5), 358–378. 10.1038/s41573-019-0012-9 30710128 PMC6927556

[B64] Wang J.-H. Gessler D. J. Zhan W. Gallagher T. L. Gao G. (2024). Adeno-associated virus as a delivery vector for gene therapy of human diseases. Signal Transduct. Target. Ther. 9 (1), 78. 10.1038/s41392-024-01780-w 38565561 PMC10987683

[B65] Westhaus A. van der Helm E. Zhang Y. Tang J. Vos Q. Pohlkamp T. (2023). AAV capsid bioengineering in primary human retina models. Sci. Rep. 13, 21594. 10.1038/s41598-023-49112-2 38081924 PMC10713676

[B66] Wu Z. Asokan A. Samulski R. J. (2006). Adeno-associated virus serotypes: vector toolkit for human gene therapy. Mol. Ther. 14 (3), 316–327. 10.1016/j.ymthe.2006.05.009 16824801

[B67] Zincarelli C. Soltys S. Rengo G. Rabinowitz J. E. (2008). Analysis of AAV serotypes 1–9 mediated gene expression and tropism in mice after systemic injection. Mol. Ther. 16 (6), 1073–1080. 10.1038/mt.2008.76 18414476

